# Anomaly Score-Based Risk Early Warning System for Rapidly Controlling Food Safety Risk

**DOI:** 10.3390/foods11142076

**Published:** 2022-07-13

**Authors:** Enguang Zuo, Xusheng Du, Alimjan Aysa, Xiaoyi Lv, Mahpirat Muhammat, Yuxia Zhao, Kurban Ubul

**Affiliations:** 1 College of Information Science and Engineering, Xinjiang University, Urumqi 830046, China; zeg@stu.xju.edu.cn (E.Z.); duxusheng@stu.xju.edu.cn (X.D.); alim@xju.edu.cn (A.A.); xmahpu76@163.com (M.M.); zhaoyuxia1128@163.com (Y.Z.); 2 Xinjiang Multilingual Information Technology Key Laboratory, Urumqi 830046, China; 3 College of Software, Xinjiang University, Urumqi 830046, China; 4 School of Mathematics and Computer Applications, Shangluo University, Shangluo 726000, China

**Keywords:** food safety risk early warning, anomaly detection, machine learning, detection data, auto-encoder

## Abstract

Food safety is a high-priority issue for all countries. Early warning analysis and risk control are essential for food safety management practices. This paper innovatively proposes an anomaly score-based risk early warning system (ASRWS) via an unsupervised auto-encoder (AE) for the effective early warning of detection products, which classifies qualified and unqualified products by reconstructing errors. The early warning analysis of qualified samples is carried out by early warning thresholds. The proposed method is applied to a batch of dairy product testing data from a Chinese province. Extensive experimental results show that the unsupervised anomaly detection model AE can effectively analyze the dairy product testing data, with a prediction accuracy and fault detection rate of 0.9954 and 0.9024, respectively, within only 0.54 s. We provided an early warning threshold-based method to conduct the risk analysis, and then a panel of food safety experts performed a risk revision on the prediction results produced by the proposed method. In this way, AI improves the panel’s efficiency, whereas the panel enhances the model’s reliability. This study provides a fast and cost-effective, food safety early warning method for detection data and assists market supervision departments in controlling food safety risk.

## 1. Introduction

With the rapid development of the internet economy, the channels available to consumers for choosing food have become more abundant, including offline dine-in and online take-out options. However, multiple channels of consumer choices place greater demands on food safety and quality prevention and control. To reduce the risk of food safety problems to human health, the proper assessment of food quality and safety risks and timely early warning is currently a controversial research issue [1]. Risk assessment-related research facilitates the evaluation of food safety risk changes and provides support for market supervision departments that aim to perform effective risk prevention and control. After the food safety law of the People’s Republic of China was issued in 2009, the China National Center for Food Safety Risk Assessment was established [2]. However, there still exists a gap between China and developed countries in the research on food quality risk assessment methods [3].

Food safety risk early warning is usually employed to identify potential hazards through risk analysis, to manage risk in the food decision-making process, and to provide scientific data support for improving food quality regulatory decision-making [4]. Therefore, establishing a good risk analysis model is the key to efficient risk early warning. Common methods for food safety risk analysis include gray relationship-based analysis [5,6], Bayesian network-based methods [7,8], machine learning-based methods [6,9,10], and artificial neural network-based methods [11,12].

**Figure 1:**
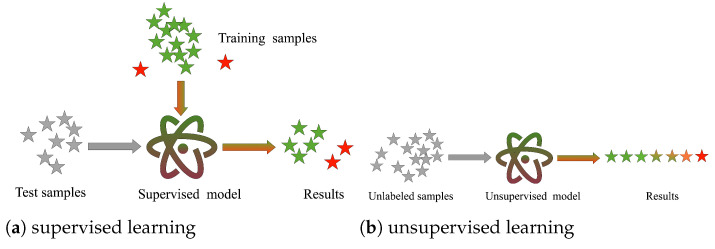
Machine learning algorithm division.

However, these methods have the following three drawbacks: (1) Their work’s training process is supervised, only focuses on two statuses of a product, qualified and unqualified, of the product, and cannot estimate the hidden dangers of the given food detection data. In the model training phase, the current method needs to artificially give or calculate the training labels and then let the model fit the training labels to make predictions for unknown samples. Nevertheless, the acquisition of risk labels increases workers’ costs and time. The difference between supervised learning and unsupervised learning is shown in Figure 1. (2) The methods need to be manually applied to perform feature engineering (complex data preprocessing), and a complex training process is necessary to utilize the raw data fully. Work such as [13,14,15] applying the risk value calculated in the first step as the expected output label for the second step of the risk model. (3) The imbalance in the data samples is not considered. As most of the “qualified” samples are not hazard samples, relation mining between “unqualified” samples and a few “qualified” samples with hazard samples is more valuable for research. To the best of our knowledge, there are no effective methods in the literature to address the sample imbalance problem.

The goal of anomaly detection (also known as outlier detection) is the process of identifying all “minorities” in a data-driven manner [16,17]. Anomaly detection is a very important subbranch of machine learning in various artificial intelligence grounded applications such as computer vision, data mining, and natural language processing. The distribution of food quality and safety inspection data is consistent with the characteristics of anomaly detection tasks, and for most of the qualified samples, the failed high-risk samples are anomalous. Thus, anomaly detection algorithms have the potential to enable food safety risk assessment. Based on this observation, in this paper, we introduce two unsupervised Auto-Encoder (AE) based anomaly detection algorithms for food safety risk assessment. The first algorithm is the classical AE [18], which has the advantages of a simple reconstruction process, stackable multiple layers, and neuroscience as a support point. In the unsupervised case, we assume that the risk samples obey different probability distributions. Because of the unbalanced food testing data samples, the trained AE can reconstruct the qualified samples for reduction but is unable to reduce the data points of the risky sample distribution better, resulting in a large reduction error. Since some of the detection metrics data are missing in food safety practical application scenarios, we further introduce the second algorithm, an improved AE, which is the denoising auto-encoder (DAE) [19]. We add Gaussian white noise to the input data so that the clean input data are partially corrupted, then feed it to the conventional AE, and let it try to reconstruct an output that is identical to the clean input. Thus, the DAE is robust to the noise in the input data.

To summarize, the main contributions are outlined as follows:

1. We propose an end-to-end unsupervised risk early warning model, which greatly improves the warning efficiency (running time) and is more realistic. Our work integrates neural network modeling into food distribution according to the principles of the hazard analysis critical control point (HACCP) system to find the key control points for risk warning and thus control the risk by conducting a comprehensive hazard analysis of each testing index.
2. Anomaly detection models are introduced for food safety risk early warning, which for the first time solve the food quality and safety warning problem from the idea of anomaly detection, quickly and efficiently solve the problem of unbalanced data samples, and provide a new possibility for food risk analysis.
3. Our proposed early warning model was verified by milk product safety detection data from a Chinese province, and extensive experiments have verified the validity of the proposed method. Noteworthy, we have mainly considered the current Chinese standard GB 25190-2010 (National Standard for Food Safety Sterilized Milk).

## 2. Related Work

### 2.1. Food Quality and Safety Risk Analysis Model Based on Machine Learning

The performance of risk assessment models is the key to food safety risk warnings. With the development of artificial intelligence, machine learning techniques are also widely employed in food safety analysis and assessment, and significant results have been achieved. Specifically, Bouzembrak et al. developed a Bayesian network model to analyze and predict chemical hazards and types of food fraud for food safety risks [7,8]. For Bayesian networks, the analysis performance is strongly influenced by experience because the network structure is usually determined by expert experience [20]. In contrast, the ANN is nonlinear and fault-tolerant and builds models that do not rely on expert experience and that can fit the data well and predict accurately [21]. As a result, ANN technology has been widely employed in the field of food safety warnings [9]. Samuel et al. utilized the fuzzy analytic hierarchy (AHP) technique to calculate the overall weight of an attribute based on its individual contribution and to predict the patient’s high-frequency risk by training an artificial neural network (ANN) classifier [11]. Wang et al. developed an early warning strategy for food transportation safety risks in real-time food safety monitoring to reduce food supply chain risks. With the development of technology, an increasing number of researchers have succeeded in improving risk models in the field of food safety early warning.

In addition, various network models, such as back propagation (BP) neural networks [18], RBF neural networks and the extreme learning machine (ELM) have been derived. Liu et al. used BP to construct an early warning model to predict whether a food product passed a test [9]. Based on the monitoring data, Zhang et al. developed a food safety early warning model using BP [10]. Geng et al. proposed a new, depth radial basis function (DRBF)-based risk warning model for sterilized milk that is combined with hierarchical analysis to model complex food safety inspection data using the concept of risk weighting [13]. However, the traditional RBF and BP converge slowly, usually requiring thousands of iterations, and the computational complexity increases rapidly when the network has many layers and nodes [22]. Compared with traditional neural networks, the ELM has a faster learning speed and higher generalization performance [23]. Therefore, the risk assessment modeling approach combined with the extreme learning machine has yielded good results [22]. Zuo et al. [24] propose using the public opinion text of food reviews as the analysis object to screen risky stores. Geng et al. propose both [25,26] used the AHP-EW algorithm to generate a combined risk value for each sample and then combined it with a machine learning model for risk prediction. On this basis, Wang et al. [27] used integrated learning techniques to improve the accuracy of the prediction models. However, existing research methods require the introduction of external expert knowledge, slow convergence, or preprocessing of food data to calculate the desired output of the model. As a comparison, the AE-based anomaly detection method discussed in this paper can concisely and quickly perform food safety risk assessment, providing new ideas for food safety risk warning.

### 2.2. Application of Anomaly Detection

With the rapid development of machine learning techniques, anomaly detection models have proliferated and achieved unprecedented results in various application areas [28]. Adewumi and Akinyelu conducted a comprehensive survey of fraud detection methods [29]. Kwon et al. extensively reviewed techniques for network intrusion detection [30]. Carter and Streilein demonstrate a probabilistic extension of exponentially weighted moving averages for anomaly detection in a streaming environment [31]. Gavai et al. compared a supervised approach developed by an expert and an unsupervised classifier with an unsupervised approach using the isolated forest method to detect insider threat tasks. Considering that this is a reasonable approach, we use the isolated forest as one of our baselines [21]. Litjens et al. presented an extensive review of the use of anomaly detection technologies in the medical field [32]. Mohammadi et al. presented an overview of techniques for the Internet of Things (IoT) and big data anomaly detection [33]. Ball et al. reviewed sensor network anomaly detection [34]. Kiran et al. introduced state-of-the-art, deep learning-based video anomaly detection methods and various classes [35]. Recently, Raghavendra et al. proposed an anomaly detection model, one class neural network (OC-NN), and applied it to graphical image anomaly detection [36]. Researchers have also applied anomaly detection-based approaches to cybersecurity tasks. Veeramachaneni et al. proposed working with neural network auto-encoders [37].

Certain successful applications in image and speech processing have utilized the data compression capabilities of AEs [38]. However, to the best of our knowledge, the current study is the first to propose the use of an AE as a food safety risk assessment model.

## 3. Materials and Methods

### 3.1. Problem Statement

In this paper, 2158 data of sterilized milk from November 2013 to October 2021 provided by the Institute of Product Quality Supervision and Inspection in Urumqi, Xinjiang Uygur Autonomous Region, China, were employed for the training to evaluate food risk. The selected raw data pertain to fresh milk. In this paper, lactose, acidity, nonfat milk solid (NMS), fat, protein, and aflatoxin M1 (AM1) are selected as detection indicators of fresh milk in this paper. The sample feature set and specific requirements are shown in Table 1.

Where $E_{1}$ is the set in which detection indicators have a minimum value limit, $E_{2}$ is the set in which detection indicators have a maximum value limit, and $E_{3}$ is the set in which detection indicators have a value limit in an interval.

In this paper, we use bold lowercase letters (e.g., **x**), bold uppercase letters (e.g., **X**), and calligraphic fonts (e.g., V) to denote vectors, matrices, and sets, respectively. Accordingly, the definitions of attributed networks are given as follows:

**Definition** **1.** *Anomaly detection on Food quality safety risk assessment.*

Given the food detection data $X\in R^{n\times m}$, where n is the number of tested samples and *m* is the number of indicators, the goal is to learn the score function f(·) to calculate the risk score $k_{i}=f\left(x_{i}\right)$ of each sample. The risk score $k_{i}$ can represent the degree of early warning of a sample $x_{i}$. By ranking all the samples with their risk scores, the anomaly risk samples can be detected according to their positions.

Note that food quality safety risk assessment via anomaly detection is performed in an unsupervised scenario.

### 3.2. ASRWS: Anomaly Score-Based Risk Early Warning System

**Figure 2:**
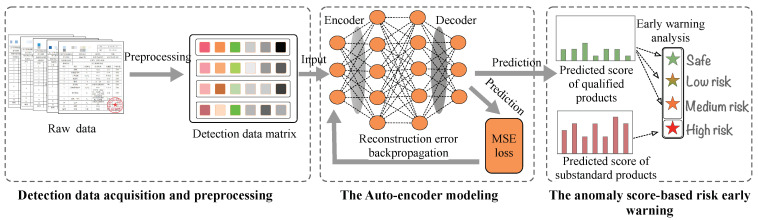
Overall architecture of ASRWS.

We propose to establish a food safety risk early screening system that uses food inspection and testing data to quickly screen products with potential safety risks. As shown in Figure 2, the ASRWS can be divided into three components: raw data processing, feature extraction, and product risk classification. The first step is to convert the raw inspection data into a data matrix that is recognizable by the feature extractor. The second step inputs the processed data into the artificial intelligence model AE or DAE utilized in this paper, and the risk value of each product is obtained through model training. In the third step, we use the risk values to classify the qualified products into three risk levels: safe, low risk, and medium risk. Note that the unqualified products are directly classified into the high-risk level. Although our proposed early screening system can significantly improve the speed and efficiency of current food safety monitoring, it cannot serve as the only method to monitor food safety, and the screened risky products need to be further evaluated by a panel of experts before they are reported to food regulatory authorities.

#### 3.2.1. Data Preprocessing

This step is the first step of the food safety risk early screening system proposed in this paper. To provide a comprehensive risk warning for food safety, the risk evaluation indicators that are selected to cover the four technical requirements of our National Standard for Food Safety of Sterilized Milk are physical and chemical indicators, contaminant limits, mycotoxin limits, and microorganisms [39]. We used Python to standardize the test values of all samples in the data preprocessing stage as follows: (1) Removal of sensory information within the test reports. We removed food sensory quality items that are not closely related to food safety, such as tissue status, color, odor, etc., to simplify the information. (2) Removal of items not detected in all samples, such as melamine. (3) Removal of redundant symbols, e.g., if a sample has a test value of “<0.2”, we remove the “<” from the result and retain the value “0.2”. Finally, the selected fresh milk data applied in this paper are shown in Table 2.

As the results of data analysis are influenced by the dimensions of different risk evaluation indices, we use the min-max normalization method to transform the original data into dimensionless data. In the comprehensive risk evaluation, a positive index indicates that the higher the index value is, the higher the risk. A negative index indicates that the higher the index is, the lower the risk [40]. Data normalization of positive indices and negative indices is achieved by Equations (1), respectively. After data normalization, the higher the data value is, the higher the risk.

(1) $$x_{i,j}^{*}=\left\{\begin{array}{c} \frac{x_{i,j}-x_{\cdot ,j}^{\min}}{x_{\cdot ,j}^{\max}-x_{\cdot ,j}^{\min}},x_{\cdot ,j}\in E_{1}\\ 1-\frac{x_{i,j}-x_{\cdot ,j}^{\min}}{x_{\cdot ,j}^{\max}-x_{\cdot ,j}^{\min}},x_{\cdot ,j}\in E_{2}\\ \frac{\left|x_{i,j}-x_{\cdot ,j}^{\mathrm{mean}}\right|}{x_{\cdot ,j}^{\max}-x_{\cdot ,j}^{\min}},x_{\cdot ,j}\in E_{3} \end{array}\right.$$

This where $x_{i,j}^{*}$ denotes the results of normalizing the data of i−th sample and j−th detection indicator. $x_{\cdot ,j}^{\max}$ is $\max\left\{x_{1},x_{2},\ldots ,x_{n}\right\}$, $x_{\cdot ,j}^{\min}$ is $\min\left\{x_{1},x_{2},\ldots ,x_{n}\right\}$ and $x_{\cdot ,j}^{\mathrm{mean}}=\frac{1}{n}\sum_{i=1}^{n}x_{i,j}$. Where $E_{1}=\left\{\mathrm{fat},\;\mathrm{protein},\;\mathrm{nonfat}\;\mathrm{milk}\;\mathrm{solids}\right\}$ is the set which detection indicators have minimum value limit, $E_{2}=\left\{\mathrm{lactose},\;\mathrm{aflatoxin}\;M1\right\}$ is the set which detection indicators have maximum value limit and $E_{3}=\left\{\mathrm{acidity}\right\}$ is the set which detection indicators have value limit in an interval.

**Figure 3:**
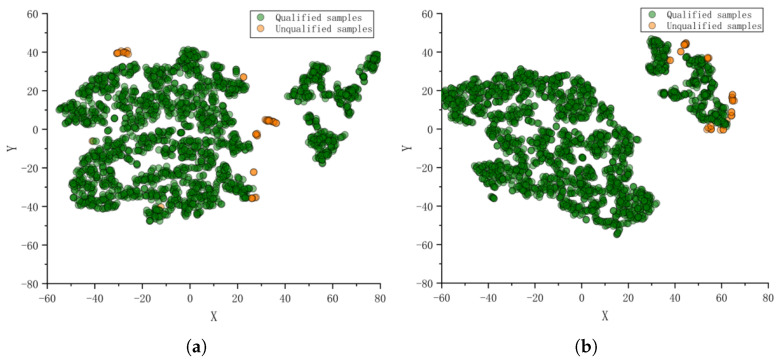
The t-SNE visualization before and after data preprocessing. (**a**) Not preprocessing. (**b**) Preprocessing.

The results of the feature visualization before and after data pre-processing are shown in Figure 3. The distribution of unqualified and qualified samples before preprocessing distribution overlap, and the unqualified samples are dispersed. As a comparison, the distribution of the pretreated failed samples is more concentrated, which is beneficial to the model detection.

#### 3.2.2. Feature Extraction

This step is the second step of the food safety risk early screening system proposed in this paper. In this paper, AE or DAE is utilized as the feature extractor of the system framework to address different scenarios in the real environment.

##### Vanilla Auto-Encoder

**Figure 4:**
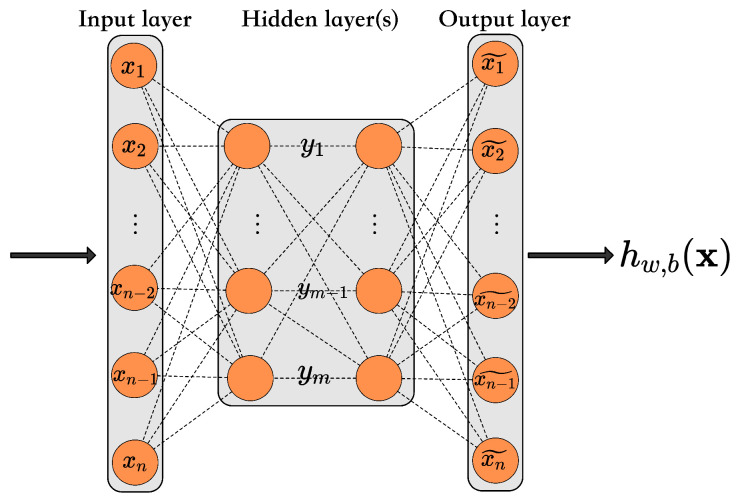
Vanilla auto-encoder.

AEs are a class of artificial neural networks that learn to encode data values in an unsupervised manner efficiently. The AE mainly consists of an encoding phase and a decoding phase and has a symmetric structure, where the encoder is used to discover a compressed representation of the given data and the decoder is used to reconstruct the original input, as shown in Figure 4.

The encoding and decoding process of the standard AE is described as follows (2)–(4):

(2) $$y=f_{\theta }\left(x\right)=\sigma \left(Wx+b\right)$$

(3) $$z=g_{\tilde{\theta }}\left(y\right)=\sigma \left(\tilde{W}y+\tilde{b}\right)$$

(4) $$z=g_{\tilde{\theta }}\left(f_{\theta }\left(x\right)\right)\approx x$$

where $x={\left(x_{1},x_{2},\ldots ,x_{n}\right)}^{T}$ belongs to n-dimensional space sample representation, $y={\left(y_{1},y_{2},\ldots ,y_{n}\right)}^{T}$ belongs to m-dimensional space new representation, $z={\left(\tilde{x_{1}},\tilde{x_{2}},\ldots ,\tilde{x_{n}}\right)}^{T}$ is output which we set equally to the input x. Parameterized by θ, $\tilde{\theta }=\left\{\left(W,b\right),\left(\tilde{W},\tilde{b}\right)\right\}$, $W\in R^{n\times m}$ and $\tilde{W}\in R^{m\times n}$ are weight matrix of the input layer and and $\left\{b,\tilde{b}\right\}$ is bias vector. σ(·) is the activation function such as Sigmoid. Therefore, the parameter optimization objective *J* is minimized the error between x and z. as shown in equal (5).

(5) $$\begin{array}{cc} J\left(\theta^{★},\tilde{\theta^{★}}\right) & =\underset{\theta ,\tilde{\theta }}{\arg\min}\frac{1}{k}\sum_{i=1}^{k}L\left(x_{i},z_{i}\right)\\ \; & =\underset{\theta ,\tilde{\theta }}{\arg\min}\frac{1}{k}\sum_{i=1}^{k}L\left(x_{i},g_{\tilde{\theta }}\left(f_{\theta }\left(x_{i}\right)\right)\right) \end{array}$$

where *L* is a loss function and we applied the squared error $L\left(x,z\right)={\sum ∥z-x∥}^{2}$. To prevent overfitting, we add a regularization term to the loss function to control the degree of weight reduction. The final AE loss function of this paper is shown in Equation (6).

(6) $$\begin{array}{cc} J_{AE}\left(\theta^{★},\tilde{\theta^{★}}\right) & =\sum E_{q_{\left(x\right)}}\left[L\left(z,x\right)\right]+\lambda ∥W∥\\ & ={\sum ∥z-x∥}^{2}+\lambda ∥W∥ \end{array}$$

where $q_{\left(x\right)}$ denotes the distribution associated with our training milk samples. λ is a hyperparameter that controls the strength of the regularization and takes values between 0 and 1. During training, the decoder forces the AE to select the most informative features, which are eventually saved in the compressed representation. The final compressed representation is in the middle coding layer. The parameters of the decoder and encoder are learned separately so that the AE tries to generate a representation that is as close as possible to its original input from the reduced-dimensional encoding.

##### Denoising Auto-Encoder

There are many samples in realistic scenarios where the detection metrics are not comprehensive, but food experts can still accurately detect risky samples. We want the risk analysis model to capture the stable structure of the input features with robustness while being useful for reconstructing the features. Inspired by this phenomenon, we select the DAE applied to milk risk analysis to add artificially and locally corrupted input $x\to \hat{x}$ to the input representation, allowing the model to learn a more robust feature representation.

**Figure 5:**
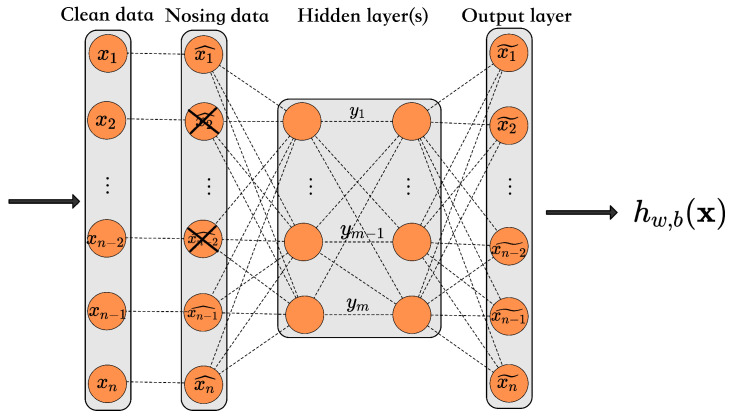
Denoising Auto-Encoder.

As shown in Figure 5, our strategy for adding noise is similar to Vincent’s strategy, where the locally corrupted input $\hat{x}$ is obtained from the clean input x by random mapping: $\hat{x}∼q_{D}\left(\hat{x}|x\right)$. The corrupted input $\hat{x}$ is then mapped in a manner similar to the vanilla AE. However, the key difference is the parameter optimization objective *J*, which makes the error between the reconstructed representation Z and the clean input *x* rather than corrupted input $\hat{x}$ as small as possible. The objective function of the DAE is shown in Equation (7).

(7) $$\begin{array}{cc} J_{DAE}\left(\theta^{★},\tilde{\theta^{★}}\right) & =\sum E_{\hat{x}∼q_{D}\left(\hat{x}|x\right)}\left[L\left(z,x\right)\right]+\lambda ∥W∥\\ & ={\sum ∥z-x∥}^{2}+\lambda ∥W∥ \end{array}$$

where $q_{D}\left(\hat{x}|x\right)$ denotes the distribution associated with our training milk samples. The optimization both AE and DAE are carried out by Adam.

In the unsupervised case, we assume that the milk risk samples obey different distributions. Because the vast majority are nonrisk samples, the trained AE preferentially reconstructs the normal samples for reduction but is unable to restore better data points that deviate from the normal distribution, resulting in a large reduction error.

## 4. Experiments and Analysis of Results

### 4.1. Evaluation Index

We have introduced three levels of indicators to determine the performance of the model in this paper. There are four primary indicators (TP, TN, FP, and FN) that represent true positives, true negatives, false positives, and false negatives, respectively. The secondary indicators use precision and recall to evaluate two different dimensions of metrics. The specific calculation method is shown in Formulas (8)–(10):

(8) $$\begin{array}{cc} \;\mathrm{Precision}\; & =\frac{TP}{TP+FP}\\ \; & =\frac{\;\mathrm{Number}\;\mathrm{of}\;\mathrm{unsafe}\;\mathrm{samples}\;\mathrm{correctly}\;\mathrm{detected}\;}{\;\mathrm{Total}\;\mathrm{number}\;\mathrm{of}\;\mathrm{samples}\;\mathrm{predicted}\;\mathrm{to}\;\mathrm{be}\;\mathrm{unsafe}\;} \end{array}$$

(9) $$\begin{array}{cc} FDR & =\frac{TP}{TP+FN}\\ \; & =\frac{\;\mathrm{Number}\;\mathrm{of}\;\mathrm{unsafe}\;\mathrm{samples}\;\mathrm{correctly}\;\mathrm{detected}\;}{\;\mathrm{Total}\;\mathrm{number}\;\mathrm{of}\;\mathrm{unsafe}\;\mathrm{samples}\;} \end{array}$$

(10) $$\begin{array}{cc} FAR & =\frac{FP}{FP+TN}\\ \; & =\frac{\;\mathrm{The}\;\mathrm{number}\;\mathrm{of}\;\mathrm{safe}\;\mathrm{samples}\;\mathrm{that}\;\mathrm{are}\;\mathrm{detected}\;\mathrm{as}\;\mathrm{unsafe}\;\mathrm{by}\;\mathrm{mistake}\;}{\;\mathrm{Total}\;\mathrm{number}\;\mathrm{of}\;\mathrm{safe}\;\mathrm{samples}\;} \end{array}$$

where precision is the inspection accuracy rate and represents the proportion of examples that are labeled hidden safety hazards among all the examples predicted to be food-safe hazards. The fault detection rate ( FDR) is the inspection completion rate and refers to the proportion of examples that are successfully obtained by the filter of all the examples labeled safety hazards. The false alarm rate ( FAR) means that safe samples are falsely detected as unsafe (the actual category is safe, and the predicted category is unsafe).

(11) $$AUC=\frac{\sum \;\mathrm{pred}{\;}_{\mathrm{safe}\;}>\;\mathrm{pred}{\;}_{\mathrm{unsafe}\;}}{\left(\mathrm{TP}+\mathrm{FN})*(\mathrm{FP}+\mathrm{TN}\right)}$$

(12) $$\;\mathrm{Accuracy}\;=\frac{TP+TN}{TP+TN+FP+FN}=\frac{TP+TN}{\;\mathrm{all}\;\mathrm{data}\;}$$

The area under the curve ( AUC) means [41] that a safe sample and unsafe sample are randomly selected from the safe sample set and unsafe sample set, respectively, and that the predicted value of the safe sample is larger than that of the unsafe sample. Formulas (11) and (12) represent the overall evaluation index and accuracy, combining the results of precision and recall.

### 4.2. Baseline Models

#### 4.2.1. K-Nearest Neighbor (KNN)

This method considers anomalies far from normal points, so for each data point, its K-nearest neighbor distance (or average distance) can be calculated and the distance can be compared to a threshold value. If the distance is greater than the threshold value, it is considered an anomaly [42].

#### 4.2.2. Local Outlier Factor (LOF)

First, for each data point, identify its K nearest neighbor value and then calculate the LOF score; the higher the score is, the more likely it is to be an outlier [43].

#### 4.2.3. Connectivity-Based Outlier Factor (COF)

The connectivity-based outlier factor is similar to the LOF, but the recorded density estimates are different [1]. In the LOF, the k-nearest neighbors are based on the Euclidean distance, which indirectly assumes that the data are distributed around the sample in a spherical fashion. However, this density estimate is problematic if the features have a direct linear correlation. The COF aims to remedy this deficiency and uses the shortest path method, which is referred to as the link distance, to estimate the local density of the neighborhood. Mathematically, this link distance is the minimum of the sum of all distances that connect all *k* neighboring samples.

#### 4.2.4. Isolation Forest (iForest)

The isolation forest basically uses a tree model to partition the data until only one individual point exists [44]. The faster the split into individual data points is, the more anomalous these data are. This result can be interpreted as points that are sparsely distributed and far from the population with high density. In statistical terms, a sparse distribution in the data space means that the probability of the data occurring in this region is low, and thus, the data falling in these regions can be considered anomalous.

#### 4.2.5. Single-Objective Generative Adversarial Active Learning (SO-GAAL)

SO-GAAL [45] is an unsupervised model based on generative adversarial networks, which can directly generate informative potential outliers based on the mini-max game between a generator and a SO-GAAL is currently a SOTA model for deep learning anomaly detection.

#### 4.2.6. K-Means

The K-means algorithm is a popular unsupervised clustering algorithm [46]. The algorithm divides the dataset into K clusters, and each cluster is represented by the mean (center of mass) of all samples within the cluster.

### 4.3. Results Analysis

#### 4.3.1. Main Results Analysis

In this section, we compare different anomaly detection methods on milk detection data to verify the performance of the method proposed in this paper. As shown in Table 3, the performance of each model is compared in all aspects by calculating multiple evaluation metrics on milk detection data. With these results, we make the following observations:

(1) The AUC and Acc values of all anomaly detection models were high, which proved that the anomaly detection algorithm could correctly predict the majority of samples. The experimental results show that the anomaly detection algorithms have good application scenarios in food safety risk analysis.
(2) The best detection results were achieved for the AE performance, except for the time spent, which was inferior to the KNN model. In particular, for the FDR metric, the AE value of 0.9024 is significantly higher than the best baseline performance of 0.8048 by 0.0976. The main reason is the ability to capture the hidden representation between the detection values of each sample, thus allowing the screening of risky samples clustered within the safe samples.
(3) In the baseline model, compared with the distance-based KNN, LOF, and COF, the ensemble-based iForest cannot achieve appreciable results, probably because certain the food risk samples are risk-free in most of the indicators, which makes it difficult to isolate their positions in the high-dimensional space with normal samples clustered.
(4) AE achieved great success on the FAR metric relative to other models, which is a significant improvement of 0.189 over the second highest KNN model of 0.3779 and an improvement of more than 100%. This finding indicates that the AE is effective in preventing risk-free samples from being incorrectly predicted as risky samples.
(5) The anomaly detection model SO-GAAL based on generative adversarial networks has the worst performance for each metric, one possible reason being that the dairy data has standard constraints for each detection metric resulting in poor quality of the pseudo data generated by the generator. From a time perspective, the clustering-based K-means takes less time, second only to KNN and AE.

#### 4.3.2. Experimental Comparison Analysis

**Figure 6:**
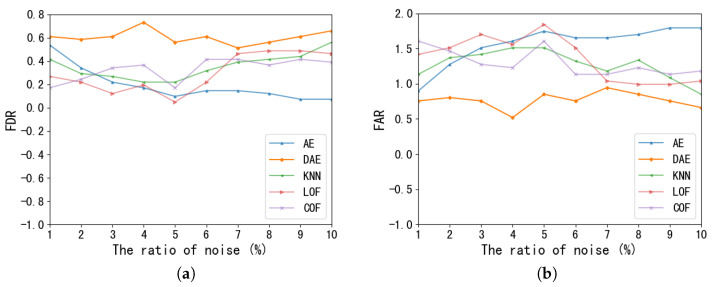
Performance of FDR and FAR for each model with different noise ratios. (**a**) FDR. (**b**) FAR.

In this section, the performance analysis of the risk completion under the intensity noise of the AE and DAE is performed to classify the risk. To assess the impact of the absence of detection data on the model prediction in the actual scenario, we artificially added noise to the AE, DAE, and LOF models for experimental comparison. Specifically, we randomly selected a certain percentage of samples to add noise to the detection value of one of their normal indicators and summarized the experimental results of adding different noise rates, as shown in Figure 6.

From Figure 6, we conclude the following points: First, one possible reason for the stable and excellent performance of the DAE model in milk anomaly detection compared with other models for different proportions of samples added to the total amount of noise is that the DAE is more robust to low-resource noise and can effectively filter the noise. In contrast, the identification of anomalous samples by AE decreases significantly as the proportion of noise increases. Second, one possible reason for the relatively low FDR values when the proportion of contaminated samples is small, i.e., when the number of samples adding noise is 3% of the total, is that when the number of contaminated samples is too small, there is a lack of sufficient information for the model to fit this missing information, resulting in a generally less robust model. Last, when the number of samples added to the noise is 5% of the total, the performance of all the models, except the AE, is improved to different degrees.

**Figure 7:**
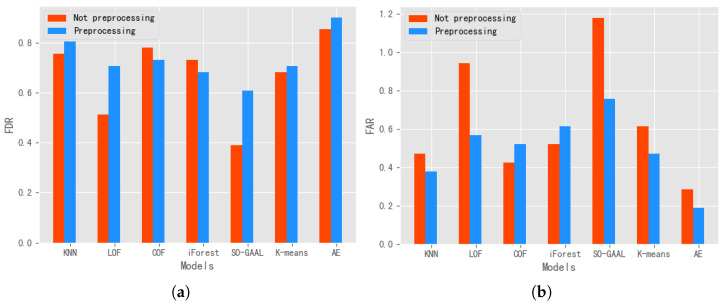
Performance of FDR and FAR for each model with preprocessing or not. (**a**) FDR. (**b**) FAR.

We also experiment with the effect of data preprocessing on each model, and the results are shown in Figure 7. It can be seen from the FDR values that, except for the COF and iForest models, other models obtained better results by processing the pre-processed data, with SO-GAAL showing the most significant improvement. Therefore, SO-GAAL is the most sensitive to data quality. From the FAR, most of the models processing the preprocessed data reduced the FAR error, with the LOF and SO-GAAL models having more significant effects. Finally, by combining the results of FDR and FAR we can prove the validity and necessity of the data standardization operation proposed in this paper.

#### 4.3.3. Visualization

**Figure 8:**
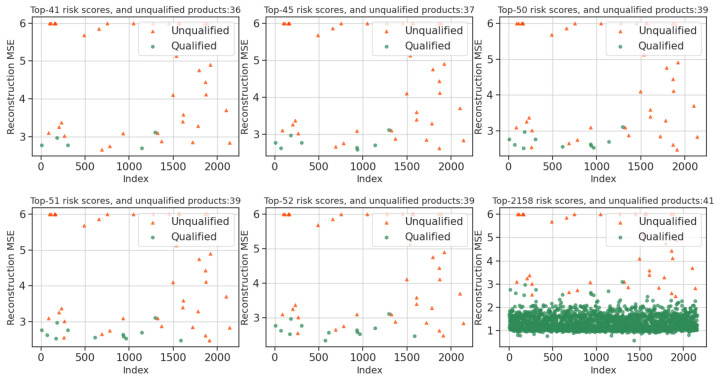
Top-n risk score visualization. *n* indicates the ranking order of risk scores. Index number means the sample order.

To visualize the effect of the AE on the risk analysis of milk products, we chose the top-n approach to visualize the risk values of all samples, as shown in Figure 8. Specifically, since there were 41 failed samples in the dataset, first, we selected the top -41 samples with the largest risk values for visualization. The resulting algorithm was able to detect 37 failed samples, with a detection rate of 90.24%. Second, we proceeded to the top-45, top -50, top-51, and top-52 samples. All the failed samples were detected by the algorithm for the top-52 samples, so we obtained the risk score threshold for this batch of samples. Last, we show the distribution of risk values for all samples, as shown in the figure for the top-2158 samples.

**Figure 9:**
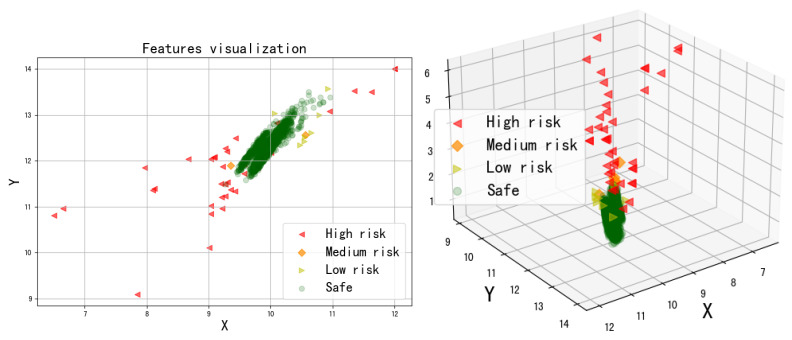
Visualization to represent four risk levels by 2D (**left**) and 3D (**right**). The ( *X*,*Y*) and *Z* denote each sample’s 2D coordinates and risk score.

The current food safety regulation only punishes unqualified samples, but qualified products are also risky. Therefore, we output the prediction results of the model and perform risk classification. As shown in Figure 9, the risk criteria are 0 (safe), 1 (low risk), 2 (medium risk) or 3 (high risk). The overall evaluation requires experts to score both the likelihood and severity of the risk. The higher the score is, the more serious the potential food safety hazard of the product. The description of each level is presented as follows:

0. $r_{qi}<r_{top-52}$**: indicates safe and no obvious food safety risk**. The qualified product risk score $r_{qi}$ is lower than the unqualified product lowest score $r_{top-52}$.
1. $r_{top-41}<r_{qi}\leq r_{top-52}$**: indicates low risk**, there is a food safety risk, but it is not apparent. The qualified product risk score $r_{qi}$ is higher than the total number of products in the unqualified product sample $r_{top-41}$ but lower than the unqualified product lowest score $r_{top-52}$.
2. $r_{qi}\leq r_{top}-41$**: indicates medium risk**, with certain food safety risks. The qualified product risk score $r_{qi}$ is higher than the total number of products in the unqualified product sample $r_{top-41}$.
3. $r_{si}\in E$** denotes high food safety risk**. The unqualified product $r_{si}$ belongs to the set of all unqualified products E.

From Figure 9, the feature representation of the samples after machine learning indicates a clear differentiation of risk levels, with samples with low-risk (safe) levels located close together and a large number of safety samples clustered together; samples with high-risk levels located far apart scattered outside the safety samples. Note that for the new input detection samples, we directly classify the risk based on the reconstruction error of the model output.

### 4.4. Effectiveness Analysis

To analyze the validity and scientificity of the risk classification proposed in this paper, we performed a t-test on the sample distribution between adjacent risk classes. Specifically, we selected 100 samples from the pool of each risk class using a randomly repeated sample survey. These samples were randomly ordered to ensure that they were blinded prior to data analysis. We summarize the p-value scores obtained by the t-test, as shown in Table 4, (1) the *p* values between the risk 3-level and the other levels are <0.05 (significant difference), which indicates a significant difference between the nonconforming products and the conforming products. (2) Increasing p-value values between each level of risk from 0 to 2 and 3-level risk indicates a synchronous trend in the distribution of qualified and unqualified samples as the risk level increases. (3) The *p* values of 1.2497 and 1.0639 for the risk {0,1} level and risk {1,2} level, respectively, are greater than 0.05, indicating no significant difference in the distribution among the warning levels of qualified samples. (4) The *p* value between risk 1-level and 2-level is smaller than the *p* value between risk 0-level and risk 1-level by 0.1858; a possible reason is that the difference between the qualified samples of risk 2-level samples increases as the risk level increases.

### 4.5. Response Measures

As suggested in [24], considering that the results directly generated by the AI model should not directly guide the work of government departments, we introduced an example analysis session by an expert panel, which manually corrects the risk warning results generated by the model. In this way, AI improves the efficiency of the expert panel, and the expert panel enhances the reliability of the model.

## 5. Conclusions and Future Work

To effectively perform early warning for testing products, we innovatively proposed an end-to-end model for early warning named the ASRWS. We use the idea of anomaly detection to classify qualified and unqualified products by the ASRWS. The early warning analysis of qualified samples is carried out by risk thresholds. The proposed method is applied to a batch of dairy product testing data from a Chinese province. The experimental results show that the unsupervised anomaly detection model can effectively analyze dairy product testing data. Extensive experiments show that the AE has higher generalization and prediction accuracy and that the DAE can effectively reduce the noise caused by missing detection values in real scenarios. Our work provides new ideas for existing research on early warning of detection data, and the unsupervised approach can significantly reduce the cost of labeling and quickly and efficiently solve problems such as unbalanced sample categories. Food safety regulatory authorities can strengthen the supervision of relevant food manufacturers based on the testing results. We will consider additional influencing factors for comprehensive risk analysis, such as environmental indices and environmental quality, in future work.

## Figures and Tables

**Table 1:** The sample feature set.

Categories		Requirements	Inspection Standard
$E_{1}$	Protein (g/100 g)	≥3.1	GB 5009.5-2010
	Fat (g/100 g)	≥3.7	GB 5413.3-2010
	NMS (g/100 g)	≥8.5	GB 5413.39-2010
$E_{2}$	Lactose (g/100 g)	≤2.0	GB 5009.8-2016
	AM1 (μg/kg)	≤0.5	GB 2761-2017
$E_{3}$	Acidity (°T)	11∼16	GB 5413.34-2010

**Table 2:** Part raw data of food inspection between 2013 and 2021. Chinese standard GB 25190-2010 (National Standard for Food Safety Sterilized Milk).

Sample ID	Date of Inspection	Inspection Item Name					
		Lactose	Acidity	NMS	Fat	Protein	AM1
20210913-761	13 September 2021	1.74	12	8.79	4.16	3.42	0.2
20180528-1284	28 May 2018	1.79	12.01	8.96	4.17	3.36	0.5
20210812-719	12 April 2021	1.73	12.2	8.8	4.1	3.42	0.2
20200409-469	9 April 2020	1.73	12.13	8.61	4.37	3.34	0.5

**Table 3:** All models run over five times with random initializations and report the mean results. Where Acc is an abbreviation for accuracy. The method with the best performance on each dataset is bolded.

Models	FDR	FAR	AUC	Acc	Time/(s)
KNN	0.8048	0.3779	0.9951	0.9925	**0.11**
LOF	0.7073	0.5668	0.9959	0.9889	9.33
COF	0.7317	0.5196	0.9956	0.9898	48.78
iForest	0.6829	0.6141	0.9931	0.9879	17.22
SO-GAAL	0.6097	0.7557	0.9879	0.9851	1.43
K-means	0.7073	0.4723	0.9947	0.9887	0.62
AE	**0.9024**	**0.1889**	**0.9963**	**0.9954**	0.58

**Table 4:** Risk level analysis. i,j denotes calculating the *p*-value between risk level *i* and level *j*, i,j∈{0,1,2,3}.

T-Test Sets	{0,3}	{1,3}	{2,3}	{0,1}	{1,2}
*p*-value	0.0381	0.0397	0.0401	1.3497	1.0639

## Data Availability

Not applicable.
